# Abdominal Tumor Mistaken for Pregnancy

**Published:** 1848-02

**Authors:** John Challice


17. *Abdominal Tumor mistaken for Pregnancy.* By John Challice, Esq. (*Lancet, Oct.* 16, 1847.)

[The following case is one of great practical value, and displays forcibly the great difficulty which surrounds the diagnosis of pregnancy. It would, perhaps, be difficult to meet with an instance affording stronger circumstantial evidence of that condition.]

Mr. Challice received an urgent message to visit a young lady, said to be laboring under cholera, but from hints received from the maid-servant he was induced to suspect the possibility of pregnancy.

When he arrived he saw a young female in bed, lying on her right side, with her face buried in the pillow, and the knees drawn up towards the abdomen. She seemed to be in pain, but was sullen, and refused to answer any questions. The mother stated that she had been vomiting, and complaining of pains in the loins, with a constant desire to pass water, and that for the last five or six months she had observed a change in her daughter—the appetite capricious, temper irritable, and on several occasions she had been surprised in tears; notwithstanding, she denied being ill, and continued to perform her domestic duties. These facts seemed confirmatory of the servant's suspicions, and with almost a conviction in his mind of the condition of the girl, the author placed his hand upon the abdomen; it was tense and swollen, and a movement like that of a living fœtus was distinctly felt; he then listened and detected a loud and quick pulsation.

The presence of these symptoms induced him to pronounce the patient pregnant. No suspicion had entered the mother's mind; she was an only daughter, and bore an excellent character. However, she did not deny the fact, but after a distressing burst of grief, and a pitiable appeal for forgiveness, she confessed that her cousin had had connection with her once, and only once, about six months before, a few days previous to his departure from England. Being unwilling to aggravate her sufferings by what appeared unnecessary enquiries, or disturb the patient by further and more careful examination, considering the case quite decisive, Mr. Challice contented himself with prescribing some simple remedy for relieving the sickness and pain. The next day there was a great improvement in the condition of the patient; the fear of discovery no longer agitated her, and she had been forgiven. Up to this period she had so contrived to compress her figure, that no increase in her bulk was perceptible when dressed, although her size was quite that of the six month of gestation when undressed. Now that this cruel mental and physical restraint no longer tormented her, she suffered less from pain and sickness, became less sullen, and more communicative.

It appears that the connection took place, after prolonged resistance, just previous to the usual period of menstruation; that up to that time there had never been the least irregularity of this function during the three years she had menstruated.

She was greatly alarmed at the absence of the accustomed appearances at the usual time, and did not feel well in health, although she had no marked symptoms; a general sense of uneasiness, with pains in the loins, and an occasional slight feeling of sickness and loss of appetite were felt. When the next period came round, she was pleased at finding herself "unwell," but only to about half the usual extent; menstruation had continued regularly up to the time Mr. Challice saw her; on each occasion, however, more and more scantily. The abdomen had gone on gradually increasing in bulk, and about five months after the connection the patient was conscious of a movement and pulsation in the abdomen, and believed herself pregnant. The breasts were small, and marked with an indistinct areola; around the eyes and mouth there were dark circles, and her mother said she had fallen away in flesh. Previous to this unfortunate occurrence, the patient not only enjoyed good health, but was remarkable for strength, endurance, and activity, inclined to *embonpoint*, full of life and spirits, and in her nineteenth year.

During the next month or six weeks Mr. Challice saw the patient occasionally. She complained of no urgent symptom, walked out now and then, had a good appetite and digestion, with sometimes slight irritability of the bladder, and irregularity of the bowels. The gradual increase in size still went on, and the mother (who now slept with the daughter) said that the movement of the child continued. The patient complained of its violence when in bed, and began to suffer from lumbar pains and constant irritation of the labia, which was much increased when she drank beer, wine, or spirits. And so the case went on.

When the ninth calendar month had nearly expired since the connection, Mr. Challice became much interested in the case, thinking it one in which the period of gestation could be accurately ascertained.

On the evening of the expiration of the ninth month the author received the expected message, with an urgent request to hasten, as very strong labor had come on. When he arrived the patient was standing at the foot of the bed, grasping the bedpost, and evidently suffering from pain, although not of a violent character. There was an interval of about ten minutes in the pains, during which she walked about the room, having a very anxious and haggard look.

After a good deal of persuasion she consented to an examination *per vaginam*, which seemed to cause excessive pain, as she screamed violently, and exclaimed that she was being murdered. At the time, the author thought the patient hysterical, but was much surprised at the narrow constricted condition of the vagina, and the presence of the hymen nearly perfect; the agony, however, produced by the examination, seemed so intolerable, that the patient, by a sudden and violent effort, threw herself from him, declaring that he should torment her no more.

Finding that the pains were weak and ineffectual, and at longer intervals, and feeling assured, from the condition of the parts, that immediate labor was not at hand, the author gave twenty minims of opium, and left, directing a full dose of castor oil to be given in a few hours. During the night she slept well; the oil acted freely in the morning; and the next day passed over without pain or any inconvenience, the patient having a good appetite, and being better in spirits. About eleven o'clock at night the pains returned with increased violence, and he found her straining and bearing down at the bedpost. An old experienced nurse declared "that the pains were quite strong enough, with *assistance*, to bring the child into the world." The mother states, that during the night she had placed her hand on her daughter's stomach, and felt the child move vigorously.

In the intervals of pain the patient walked about the to room, and was cheerful, except expressing what seemed be an unreasonable horror at any examination. The pains commenced in the abdomen, and then extended round to the loins, came on regularly every ten minutes, and were marked with all the characteristics of labor in first stage.

The extreme excitement and dread which the patient evinced when the necessity for an examination was impressed upon her induced the author to waive it, although he was anxious to ascertain the real condition of affairs. It would be useless to detail the diurnal symptoms; suffice it that a week passed over, and matters remained apparently without alteration either one way or the other. I may here state that menstruation did not take place at this period. Doubts now began to rise in the author's mind about the nature of the case; and, when nine calendar months from the departure of her cousin had expired he became very anxious about it. It was at this stage that Dr. Lever was consulted. After a careful and thorough external and internal examination, this gentlemen, justly famous for his skill and tact in diagnosis, having the history of the case before him, came to the conclusion that it was

"extra-uterine impregnation." At that time her physical condition was as follows:—Countenance pale, an anxious expression; eyes rather sunken; nose pinched; breasts somewhat flaccid; abdomen the size of mature pregnancy, if not larger; bowels sometimes costive for a day or two, at others times the reverse; urine most frequently pale and copious, but on some occasions thick, scanty, and high-colored. Over the entire abdominal region a distinct pulsation could be heard and felt; but owing to the extreme excitability of the patient it was almost impossible to ascertain whether or not it was cynchronous with the pulse. Palliative measures were adopted, and the case, now become one of painful interest, was closely watched. During the next fortnight no perceptible alteration occurred, except that the pulsation in the tumor became less distinct, and the abdomen more tense. Dr. Ferguson now visited the patient, and pronounced the abdominal pulsation to be synchronous with the heart's action, and doubted whether impregnation had taken place at all. On his recommendation the author punctured the abdomen with a fine "trochar," and drew off about five pints of thick grumous and offensive matter. Great relief followed the operation, only, however, temporary; for in the course of a short time the abdomen became as tense as before, and all the patient's sufferings returned. The interest, in a further detail of the symptoms of this case, here ceases, no doubt now being entertained of its character. After a second and third tapping, the poor girl gradually got weaker and weaker, her only comfort the oblivion produced by anodynes; and on the 15th of February she died.

The day following assisted by Mr. Druitt, a post-mortem examination was made. The upper portion of the body was extremely emaciated, but owing to slight œdema of the lower extremities, this appearance was not general. Abdomen greatly distended, and marked by enlarged veins; it measured in circumference fifty-eight inches. About a gallon of fluid was drawn off by the trochar, previous to making a free incision, after which nearly a pailful of brain-like matter rolled out. This had been contained in a cyst, which extended from the pubis to the ensiform cartilage, and from the left to the right hypochondrium; in some parts the walls of the sac were more than an inch thick, and of a fibro-cartilaginous consistence; the anterior portion adhered firmly to the abdominal parieties, the upper being formed by the inferior surface of the liver; that organ was bathed with the contents of the sac, and became inoculated, several small cysts, filled with medullary sarcoma, having formed in its substance. There were, also, many isolated cysts, varying from the size of a hazel nut to that of a pigeon's egg, formed in the walls of the cyst; these had no connection with each other, or communication with the general cavity. The uterus was found imbedded in the lowor portion or base of the cyst; no trace of the ovaries could be met with; the bladder was small, but not affected by disease.

The peculiar interest of this case arises from the close resemblance to the symptoms of impregnation; the development of a malignant disease seeming, in a great measure, to be influenced by the feelings or instinct of the patient. The author asks, would the girl have died had no connection taken place? How far did the mental and physical excitement act upon the origin or the progress of the disease? Or was it completely independent and its course inevitable?

[It is not improbable that the ovarian excitement, connected with the act of copulation, was the starting point of the disease.]

